# mtDNA, Metastasis, and the Mitochondrial Unfolded Protein Response (UPR^mt^)

**DOI:** 10.3389/fcell.2017.00037

**Published:** 2017-04-19

**Authors:** Timothy C. Kenny, Doris Germain

**Affiliations:** Division of Hematology/Oncology, Department of Medicine, Icahn School of Medicine at Mount Sinai, Tisch Cancer InstituteNew York, NY, USA

**Keywords:** metastasis, mitochondrial DNA, mitochondrial unfolded protein response, oxidative stress, breast cancer

## Abstract

While several studies have confirmed a link between mitochondrial DNA (mtDNA) mutations and cancer cell metastasis, much debate remains regarding the nature of the alternations in mtDNA leading to this effect. Meanwhile, the mitochondrial unfolded protein response (UPR^mt^) has gained much attention in recent years, with most studies of this pathway focusing on its role in aging. However, the UPR^mt^ has also been studied in the context of cancer. More recent work suggests that rather than a single mutation or alternation, specific combinatorial mtDNA landscapes able to activate the UPR^mt^ may be those that are selected by metastatic cells, while mtDNA landscapes unable to activate the UPR^mt^ do not. This review aims at offering an overview of the confusing literature on mtDNA mutations and metastasis and the more recent work on the UPR^mt^ in this setting.

Using cytoplasmic hybrid (cybrid) technology, Ishikawa and colleagues were the first to demonstrate that mitochondiral DNA (mtDNA) mutations could alter the metastatic behavior of cancer cells (Ishikawa et al., 2008b). In their seminal work, they demonstrated that the replacement of endogenous mtDNA from a non-metastatic cell line with mtDNA from a highly metastatic cell line was able to confer metatastic behavior *in vivo* (Ishikawa et al., 2008b). The ability of metastatic mtDNA to induce metastasis in cells with non-metastatic nuclei was linked to a specific ROS-generating mutation in the ND6 gene of the mitochondrial genome (G13997A and 13885insC) (Ishikawa et al., 2008b). Ishikawa and colleagues showed that these mutations lead to a defect in complex I activity leading to the production of reactive oxygen species (ROS) (Ishikawa et al., 2008a,b; Ishikawa and Hayashi, 2009). Further, they showed that mtDNA mediated metastasis was dependent on the production of ROS, as pretreatment of tumor cells with the ROS scavenger *N*-acetylcysteine (NAC) supressed metastasis in mice (Ishikawa et al., 2008a,b; Ishikawa and Hayashi, 2009). The authors postulated that metastatic mtDNA may mediate its pro-invasion effects through the differential expression of nuclear-encoded genes and provided evidence for the upregulation of some metastasis-related genes such as MCL-1, HIF-1α, and VEGF (Ishikawa et al., 2008a,b; Ishikawa and Hayashi, 2009). The potential role of inter-organelle communication between the mitochondria and the nucleus in explaining their findings is an intriguing possibility. However, up to now mechanistic evidence supporting this hypothesis was lacking.

Subsequently, the Hayashi group demonstrated the ability of mtDNA to influence metastasis using the highly metastatic breast cancer cell line, MDA-MB-231, to generate two cybrids—one with nuclei from MDA-MB-231 cells paired with mitochondria of MDA-MB-231 cells (231mt231) and the other with nuclei from MDA-MB-231 cells paired with the normal mitochondria of fetal fibroblasts (231mtFt). Comparing these two cybrids, they showed that despite sharing the same nucleus from a metastatic cell line, only cells with mitochondria from MDA-MB-231 cells (231mt231) were highly metastatic and exhibited complex I defects (Imanishi et al., 2011; Ishikawa et al., 2012). Pathogenic point mutations in the ND4 gene (C12084T) and ND5 gene (A13966G) present in the mtDNA of MDA-MB-231 were shown to be responsible for the described differences (Imanishi et al., 2011; Ishikawa et al., 2012). As expected, replacement of pathogenic MDA-MB-231 mtDNA with fetal fibroblast mtDNA, which is devoid of these mutations, abolished complex I deficiency, and reduced metastatic potential (Imanishi et al., 2011; Ishikawa et al., 2012).

Following the initial work by Hayashi's group, other laboratories have reported similar findings, further substantiating the link between mtDNA and metastasis. Notably, also using cybrids, the pathogenic mtDNA mutation A12308G in the tRNA^Leu(CUN)^ gene found in MDA-MB-435 cells was shown to regulate metastasis *in vivo* (Kulawiec et al., 2009). Further, using genetic approaches to control the activity of complex I of the electron transport chain, evidence of the ability of mtDNA mutations to control metastatic behavior of cancer cells was obtained (Santidrian et al., 2013). Specifically, the knockdown of the subunit NDUFV1, which causes complex I dysfunction, promoted metastasis (Santidrian et al., 2013). As many of the subunits of complex I are encoded in the mitochondrial genome, mtDNA mutations or polymorphisms in these genes could contribute to complex I function and influence cancer progression. Looking at the influence of mtDNA mutations on tumor formation of cybrids in nude mice, another group demonstrated that mild mtDNA mutations (G3460A in ND1, G11778A in ND4, and T14484C in ND6) showed enhanced tumorigenicity over severe mtDNA mutations (G8363A in tRNA^Lys^) (Cruz-Bermúdez et al., 2015). Also using cybrids to compare 143 osteosarcoma cells with wild-type or tRNA^Leu^ (A3243T) mutant mtDNA, Nunes and colleagues showed increased cell motility and invasion in cybrids with mutant mtDNA (Nunes et al., 2015). These *in vitro* findings were substantiated *in vivo* with mice injected with cybrids with mutant mtDNA exhibiting increased lung metastases (Nunes et al., 2015). Further, since the cybrids with mutant mtDNA showed oxidative phosphorylation (OXPHOS) dysfunction, the authors postulated that defects in mitochondria may affect the extracellular matrix, resulting in increased migratory capacity (Nunes et al., 2015).

Additional *in vivo* evidence on the influence of mtDNA in cancer progression has been obtained from Mitochondrial-Nuclear eXchange (MNX) mouse models of breast cancer (Feeley et al., 2015). MNX mice were generated with a FVB/NJ nuclear genetic background with either C57BL/6J, BALB/cJ, or FVB/NJ mtDNA and then crossed to introduce the PyMT oncogene to form spontaneous mammary tumors (Feeley et al., 2015). Using this mouse model, Feeley and colleagues then assessed the rate of tumor formation and progression in mice with identical nuclear backgrounds but different non-pathogenic mtDNA backgrounds under the same oncogenic driver (Feeley et al., 2015). In this study, the authors found that a significant difference in breast cancer tumor formation and metastasis between MNX strains with mice carrying BALB/cJ mtDNA having accelerated primary tumor onset and enhanced metastatic dissemination (Feeley et al., 2015). These findings are striking considering the minor differences in mtDNA sequence between these strains. Notably, the FVB/NJ and C57BL/6J differ by a missense mutation in subunit II of complex IV and a polymorphism in subunit III of complex I, while FVB/NJ, and BALB/cJ differ by missense mutations in subunit II and III of complex IV (Feeley et al., 2015). The ability of polymorphic mtDNA mutations to influence tumor progression was also demonstrated by Hayashi's group using mouse cybrids with nuclear DNA from C57BL/6 mice and mtDNA from C3H/an mice (Takibuchi et al., 2013). Cybrids were generated from poorly metastatic Lewis lung carcinoma P29 cells collected from a C57BL/6 mouse and fused with mtDNA from the C57BL/6 mice or C3H/An mice (Ishikawa et al., 2010; Takibuchi et al., 2013). In these studies, cells with C3H/An mtDNA were more invasive when assessed by invasion assays than syngenic mouse cells with C57BL/6 mtDNA, further emphasizing the ability of nonpathogenic mtDNA backgrounds to influence cancer progression (Takibuchi et al., 2013).

Some clinical evidence also supports the notion of a role of mtDNA in cancer progression. First, the LaFramboise group demonstrated using next-generation sequencing on breast cancer samples from 99 women, that 73.7% of patient tumors contained somatic mtDNA mutations, the majority of which were found in genes encoding subunits of complex I (McMahon and LaFramboise, 2014). By sequencing primary lung adenocarcinoma samples from patients, Yuan and colleagues identified missense and nonsense mtDNA mutations in the ND6 gene in some patients which correlated positively with worse pathological grade and stage and with the presence of lymph node metastases (Yuan et al., 2015). Further expanding their findings, they generated cybrids with the nucleus of a lung adenocarcinoma cell line (A549) and patient derived mtDNA with missense or nonsense mutations in ND6. They reported that both missense and nonsense mtDNA mutations increased ROS production and tumor cell migration and invasion with respect to wild-type mtDNA controls (Yuan et al., 2015). In a case-control study of Chinese women with breast cancer, women with mtDNA macro-haplotype N were more likely to have metastatic disease than women with macro-haplotype M (Fang et al., 2010). Despite adjusting for co-variables such as age, BMI, and hormone receptor status, macro-haplotype N women continued to have significantly increased rates of metastasis (OR = 0.39; 95%CI 0.17–0.94; *p* = 0.036) (Fang et al., 2010). Of particular note is that the macro-haplotypes M and N are defined by a single nucleotide polymorphism at position 10,400 in the ND3 gene. In a clinical cohort of patients with hepatocellular carcinoma, another study identified two polymorphisms in the D-loop of mtDNA (315insC and 16263 T/C) that were independent predictors of tumor-free survival time (Li et al., 2016). Increased tumor-free survival time was seen in patients with the 16263T allele compared to the 16263C allele and in patients with a C insertion at 315 compared to those without the insertion (Li et al., 2016).

The body of literature surrounding mtDNA and its role in cancer progression and metastasis points to an indisputable link between the mitochondrial genome and cancer progression. Importantly, clinical evidence argues that the influence of mtDNA on cancer progression is relevant to human disease and deserving of further investigation. However, despite the significant amount of evidence supporting a link between mtDNA and metastasis, the nature of mutations or alterations responsible for this connection remains ambiguous. Initial reports identified the ability of severe missense mutations in protein encoding regions of the mitochondrial genome to promote metastasis (Ishikawa et al., 2008a,b, 2012; Ishikawa and Hayashi, 2009; Imanishi et al., 2011). Others have demonstrated that mutations in non-protein coding regions of the mitochondrial genome also influence metastasis (Kulawiec et al., 2009; Cruz-Bermúdez et al., 2015). Further complicating our understanding of the types of mutations able to influence metastasis, one group reported that minor missense mutations in mtDNA were actually more potent at promoting metastasis than major missense mutations (Cruz-Bermúdez et al., 2015). Moreover, nonpathogenic mutations, synonymous mtDNA variants, and even human macro-haplotype differences in mtDNA have been shown to influence cancer metastasis and clinical outcomes (Fang et al., 2010; Takibuchi et al., 2013; Feeley et al., 2015; Yuan et al., 2015; Li et al., 2016). Taken together, these reports offer conflicting accounts as to the nature and severity of mtDNA mutations able to exert effects on cancer cell metastasis. Additionally, the mechanisms by which these mtDNA mutations and polymorphisms influence metastasis are not well described or understood at the molecular level. In their original paper, the Hayashi group hypothesized that differential nuclear gene expression in response to mtDNA mutations and subsequent mitochondrial dysfunction could be one mechanism by which mtDNA could influence metastasis (Ishikawa et al., 2008b). Consistent with that possibility, multiple studies have demonstrated the ability of mtDNA and mitochondrial function to influence global nuclear gene expression patterns and activate large transcriptional programs to coordinate directed changes in the cell (Hwang et al., 2011; Jandova et al., 2012; Guantes et al., 2015).

One potential mechanism of communication between the mitochondria and the nucleus is the activation of the mitochondrial unfolded protein response (UPR^mt^). Originally identified in mammalian cells using the overexpression of misfolded ornithine transcarbamylase (OTCΔ) in the mitochondrial matrix, the UPR^mt^ leads to the activation of the transcription factor CHOP, which in turn promotes the transcription of proteases, such as ClpP, and chaperones, such as HSP60, to respond to the proteotoxic stress in the mitochondria (Martinus et al., 1996; Zhao et al., 2002; Figure 1). The promoter of HSP60 has been used extensively in *C. elegans* as a readout of the UPR^mt^ and has formed an impressive body of literature that has expanded our understanding of the UPR^mt^ in worms (Benedetti et al., 2006; Haynes et al., 2007, 2010; Durieux et al., 2011; Nargund et al., 2012; Mohrin et al., 2015; Merkwirth et al., 2016; Tian et al., 2016). Most notable from this work was the identification of ATFS-1 as the mechanistic mediator of the UPR^mt^ in worms (Haynes et al., 2010; Nargund et al., 2012). It has recently been reported that the mammalian ortholog of ATFS-1 is the transcription factor ATF5 (Fiorese et al., 2016). As ATF5 has been described downstream of CHOP activation (Teske et al., 2013), this recent discovery places ATF5/ATFS-1 under the CHOP axes of the UPR^mt^ originally described by Hoogenrad's group (Zhao et al., 2002) and establishes the conservation of this pathway in worms (Fiorese et al., 2016; Figure 1).

**Figure 1:**
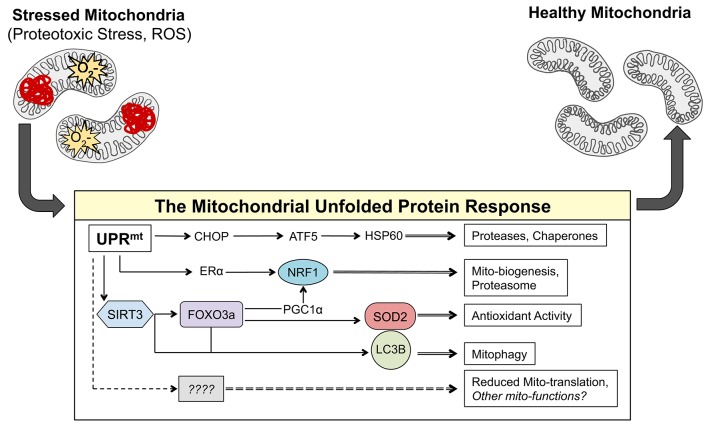
**The axes of the UPR^mt^ work in parallel to result in mito-protective effects**. Accumulation of misfolded proteins in the mitochondria and/or ROS activates currently known transcription factors CHOP, ATF-5, NRF1, ERα, FOXO3a, and most likely many others. These transcription factors induce the expression of gene products that result mito-protective effects to return the mitochondrial network to a healthy state.

In addition to CHOP, work from our group has identified two additional and independent axes of the UPR^mt^ in mammals that are activated in response to proteotoxic stress (Papa and Germain, 2011, 2014). The estrogen receptor alpha (ERα) axis of the UPR^mt^ is activated in response to proteotoxic stress in the inter membrane space of the mitochondria and leads to the transcription of OMI/HTRA2, NRF1, and activation of the proteasome (Papa and Germain, 2011; Figure 1). The other axis of the UPR^mt^ our group has described is regulated by the mitochondrial sirtuin SIRT3 and involves the activation of mitochondrial antioxidant genes and removal of irreversibly damaged mitochondria through mitophagy (Papa and Germain, 2014; Figure 1). The SIRT3 axis of the UPR^mt^ activates FOXO3a, which subsequently results in the induction of manganese superoxide dismutase (SOD2) (Papa and Germain, 2014). Notably, a Sirtuin/FOXO/SOD2 pathway has also been described in *C. elegans* and found to extend lifespan (Mouchiroud et al., 2013). While SIRT3 was found to be up-stream of FOXO3a, since inhibition of SIRT3 by shRNA abolished the activation of FOXO3a under proteotoxic stress conditions (Papa and Germain, 2014), how a sirtuin in the matrix of the mitochondria leads to activation of FOXO3a in the cytoplasm remains unclear and is likely indirect.

The complex and multifactorial transcriptional response that is activated by the axes of the UPR^mt^ in response to proteotoxic and oxidative stress in the mitochondria has recently been characterized using an omics approach (Münch and Harper, 2016). This work highlighted the attenuation of mitochondrial translation as a downstream effect of the activation of the UPR^mt^ (Münch and Harper, 2016; Figure 1). The complex and well-orchestrated changes in nuclear gene expression induced by the UPR^mt^ described in this study provide further circumstantial evidence that the UPR^mt^ may mediate retrograde signaling between the mitochondria and the nucleus in response to mitochondrial dysfunction. Increased ROS in the mitochondria can lead to the oxidation of proteins causing their misfolding and aggregation. Such oxidative and proteotoxic stress in the mitochondria leads to the activation of the UPR^mt^, further placing the UPR^mt^ as a potential mechanism to explain the ability of mtDNA to influence metastasis.

Additional evidence connecting the UPR^mt^ and mtDNA come from studies focused on mitochondrial disease and mechanisms by which deleterious mtDNA strains are propagated. Mitochondrial disease, often a result of mutations in protein coding regions of mtDNA, can result in OXPHOS dysfunction and subsequent activation of the UPR^mt^. In the *C. elegans* model system, the Haynes group demonstrated that mtDNA mutations that result in OXPHOS defects activate the UPR^mt^ and are inadvertently propagated by the cell in an attempt to restore OXPHOS function (Lin et al., 2016). In agreement with this observation, another recent report demonstrated that mutant mtDNA exploits mtDNA copy-number control homeostasis and the UPR^mt^ to “hitchhike” to high frequency in cells (Gitschlag et al., 2016). In their model, high levels of mutant mtDNA lead to insufficient energy output and mitochondrial stress, which activate the UPR^mt^ to promote mitochondrial biogenesis and mtDNA replication (Gitschlag et al., 2016). UPR^mt^-mediated mtDNA replication is not selective to non-mutant mtDNA and therefore facilitates the propagation of mutant mtDNA as well, which only further exacerbates the problem (Gitschlag et al., 2016).

The Germain group identified the ERα and SIRT3 axes of the UPR^mt^ (Papa and Germain, 2011, 2014) in breast cancer cells. Recent work in our lab has sought to understand the link between mtDNA and cancer metastasis and provided evidence for a role of the SIRT3 axis of the UPR^mt^ in facilitating disease progression (Kenny et al., 2017). We initiated this study using a panel of invasive breast cancer cells lines (MDA-MB-231, MDA-MB-361, and MDA-MB-157) and non-invasive cell lines (MCF7, MCF7R, and ZR75.1). Sequencing of the mtDNA of these cells revealed no consensus in mtDNA mutations that could differentiate invasive from non-invasive cell lines. Additionally, no correlation was found between the number or position of mtDNA mutations/polymorphisms and invasion capacity. A common characteristic of all invasive cells compared to non-invasive cells, however, was the presence of at least one mtDNA variant with high levels of heteroplasmy. As each cell contains several copies of mtDNA, if all copies carry the same sequence, this is referred as homoplasmy. However, if different mtDNA copies carry different sequences, including mutations in coding sequences or variants in non-coding regions of mtDNA, this is referred as heteroplasmy. Additionally, heteroplasmic cell lines had heterogeneous morphology by transmission electron microscopy and demonstrated metabolic flexibility as defined by their preference for oxidative phosphorylation or glycolysis (Kenny et al., 2017).

We then tested if activation of the UPR^mt^ could differentiate invasive from non-invasive cells. Interestingly, no differences in HSP60 levels were seen across the cell line panel, suggesting that the CHOP axis of the UPR^mt^ does not mediate the observed differences in invasion. However, it is important to note that while no changes in HSP60 were observed in established cancer cell lines, we also found that HSP60 may be up-regulated early during transformation. In contrast, all markers of the SIRT3 axis of the UPR^mt^ were activated in invasive cells and not in non-invasive cells.

To directly address the influence of mtDNA alone on induction of the SIRT3 axis of the UPR^mt^, we took advantage of a collection of cybrids derived from the 143B/206 osteosarcoma cell line devoid of mitochondria. These cybrids were constructed using either wild-type non-pathogenic mtDNA (WT), pathogenic mtDNA from a patient with a frame-shift mutation in cytochrome B (CyB), or a hybrid background with mtDNA from the patient with the frame-shift mutation in cytochrome B in combination with the mtDNA from another patient (Hybrid B).

We found that both CyB and Hybrid B were significantly more invasive than WT cells with non-pathogenic mtDNA and activated markers of the SIRT3 axis of the UPR^mt^ compared to WT cells. Some subtle differences in invasion and activation of the SIRT3 axis of the UPR^mt^ were also noted between the CyB and Hybrid B. These differences correlated with minor differences in heteroplasmy between the two cell lines. Therefore, despite the fact that CyB and Hybrid B cells contain the same nucleus and have the same mtDNA frameshift mutation in CyB, their relative invasion capacity and induction of the SIRT3 axis of the UPR^mt^ differs, suggesting that the minor differences in mtDNA heteroplasmy are responsible for these differences (Kenny et al., 2017).

To validate the relevance of the SIRT3/FOXO3a/SOD2 axis of the UPR^mt^ to human disease we analyzed a collection of primary breast cancer samples from patients. Our data suggest that patients with tumors positive for the SIRT3 axis of the UPR^mt^ have significantly worse disease free survival. Further, using patient matched primary and metastatic lesions, we found a significant enrichment of UPR^mt^ positive sections in the metastatic lesions. These results support the notion that cells able to activate the SIRT3 axis of the UPR^mt^ are more invasive and are therefore clonally selected for during metastatic progression (Kenny et al., 2017).

We propose that these most recent data may reconcile the conflicting literature regarding the nature of the mtDNA mutations/alterations responsible to increase metastatic potential of cancer cells. Our data suggest that the ability of a single mtDNA alteration to influence metastasis is largely based on the surrounding mitochondrial genomic landscape. In this setting, some combinations of mtDNA alterations activate the UPR^mt^, while others do not. These mtDNA landscapes able to activate the UPR^mt^ confer cells increased mitochondrial fitness, which in turn may positively influence invasion capacity and metastasis. Clearly, the UPR^mt^ is a complex transcriptional program that extends well beyond anti-oxidant, autophagy and proteases. However, the rising evidence of a link between mtDNA, the UPR^mt^, and metastasis already holds the promise that the UPR^mt^ could be exploited therapeutically in our fight against cancer. However, one very important question that remains to be answered is the possibility that metastases at different anatomical sites may select for different mtDNA landscapes and activation of different axis of the UPR^mt^ as possible differences may be influenced by the different microenvironment specific to these various tissues. Future work will be required to answer the many questions regarding this novel pathway.

## Author contributions

TK has reviewed the literature and wrote the first draft of the manuscript. DG has generated the final version.

### Conflict of interest statement

The authors declare that the research was conducted in the absence of any commercial or financial relationships that could be construed as a potential conflict of interest.
